# Optically controlled electroresistance and electrically controlled photovoltage in ferroelectric tunnel junctions

**DOI:** 10.1038/ncomms10808

**Published:** 2016-02-29

**Authors:** Wei Jin Hu, Zhihong Wang, Weili Yu, Tom Wu

**Affiliations:** 1 Materials Science and Engineering, King Abdullah University of Science and Technology (KAUST), Thuwal 23955-6900, Saudi Arabia; 2 Advanced Nanofabrication Core Lab, King Abdullah University of Science and Technology (KAUST), Thuwal 23955-6900, Saudi Arabia

## Abstract

Ferroelectric tunnel junctions (FTJs) have recently attracted considerable interest as a promising candidate for applications in the next-generation non-volatile memory technology. In this work, using an ultrathin (3 nm) ferroelectric Sm_0.1_Bi_0.9_FeO_3_ layer as the tunnelling barrier and a semiconducting Nb-doped SrTiO_3_ single crystal as the bottom electrode, we achieve a tunnelling electroresistance as large as 10^5^. Furthermore, the FTJ memory states could be modulated by light illumination, which is accompanied by a hysteretic photovoltaic effect. These complimentary effects are attributed to the bias- and light-induced modulation of the tunnel barrier, both in height and width, at the semiconductor/ferroelectric interface. Overall, the highly tunable tunnelling electroresistance and the correlated photovoltaic functionalities provide a new route for producing and non-destructively sensing multiple non-volatile electronic states in such FTJs.

## Abstract (web summary)

Tunnelling electroresistance is the variation of resistance of a thin-film junction with the polarization state of its ferroelectric tunnel barrier. Here the authors demonstrate a large light-modulated tunnelling electroresistance and a hysteretic photovoltaic effect in a complex oxide heterostructure.

## Graphical abstract

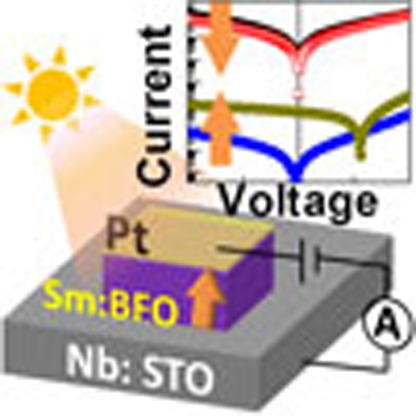

A ferroelectric layer sandwiched between two metal electrodes serves as the basic building block in diverse applications, such as ferroelectric capacitors^1^,^2^,^3^ and ferroelectric diodes^4^,^5^, which utilize polarization as the switching degree of freedom to perform logic and memory functions. As the thickness of the ferroelectric layer decreases to a few nanometres, electrons can quantum mechanically tunnel through the ferroelectric barrier, which is the basis for the operation of so-called ferroelectric tunnel junctions (FTJs)^6^,^7^. In fact, the concept of FTJ was formulated by Esaki *et al.*^8^ more than four decades ago, but it has only recently aroused considerable interest following the demonstration of ferroelectricity in ultrathin perovskite films with a thickness of several unit cells^9^,^10^,^11^.

A basic feature of FTJs is the modulation of conductance between on and off states with the reversal of ferroelectric polarization, namely, the tunnelling electroresistance (TER)^6^,^7^. Tsymbal and Kohlstedt^12^ theoretically demonstrated that polarization-related physical factors, such as interface charge screening, interface atomic bonding and the inverse piezoelectric effect, can change the interfacial barrier height and/or width, thereby leading to the TER effect. Most FTJs with metallic electrodes exhibit TER values on the order of ∼1–10^3^, and the effect is attributed to the modulated ferroelectric barrier height^13^,^14^,^15^,^16^,^17^,^18^,^19^,^20^,^21^. Recently, a giant TER of up to 10^4^ was observed in a FTJ of Pt/BaTiO_3_ (BTO)/Nb:SrTiO_3_ (NSTO)^22^. This remarkable improvement in performance originates from the suppression/enhancement of the space charge layer in the semiconducting NSTO electrode, that is, both the height and width of the ferroelectric tunnel barrier were modulated^22^,^23^. Enhancement of TER was also reported in La_0.7_Sr_0.3_MnO_3_/BTO/La_0.5_Ca_0.5_MnO_3_/La_0.7_Sr_0.3_MnO_3_(LSMO) tunnel junctions (TER∼100 at 5 K) (ref. 18) and PbZr_0.2_Ti_0.8_O_3_/La_1−*x*_Sr_*x*_MnO_3_ heterostructures (TER∼300 at 300 K) (ref. 24), which were attributed to the ferroelectric-induced phase modulation of the manganite electrode. Recently, it was recognized that ultrathin ferroelectric films are in poly-domain states with nm-scale domains^25^, which is a direct result of the fact that the ferroelectric domain size scales with the square root of the film thickness^26^. This insight offers the opportunity of engineering the domain states and creating multiple resistive states with memristive behaviour in FTJs based on ultrathin BTO (ref. 27) and tetragonal BiFeO_3_ (BFO; ref. 28) films. FTJ-based memristor was also demonstrated in Co/BTO/LSMO heterostructures, and the operating mechanism was related to the field-induced interface charge redistribution^29^. These ground breaking results provided new routes towards the development of high-performance FTJs, and thus, the underlying mechanism clearly warrants further investigations.

Concurrently, the demands for higher density, low-power data processing/storage devices have motivated the development of oxide heterostructures that possess integrated functionalities and that can be manipulated by multiple external parameters^30^,^31^,^32^,^33^,^34^,^35^. Effective approaches towards integrating novel functionalities into FTJs are, however, sparse^36^. The most notable approach is the multiferroic tunnel junction, in which ferroelectric tunnel layers are combined with ferromagnetic electrodes and in which magnetic properties such as magnetoresistance and spin polarization can be controlled by an electric field in addition to a magnetic field^16^,^17^,^18^. Because some perovskite oxides are known to be good light absorbers^31^,^37^,^38^, light illumination could be envisioned as an additional tuning parameter for controlling the operation of FTJs.

Herein, we report complimentary electrical and optical control over the resistive switching in FTJs of Pt/Sm_0.1_Bi_0.9_FeO_3_(SBFO)/NSTO. A colossal TER of greater than 10^5^ at room temperature has been observed in our FTJs, which could be further modulated by ∼10-fold through light illumination. Our detailed analysis suggests that the colossal TER originates from the electrically induced transition between the conduction modes of direct tunnelling and Schottky thermionic emission at the ferroelectric/semiconductor interface. In addition, the light-absorbing depletion layer in NSTO induces a photovoltaic (PV) effect, which is dependent on the resistance state of the device and provides a new route to non-destructive reading in addition to the conventional current-based route.

## Results

### Device structure and band alignment

**Figure 1:**
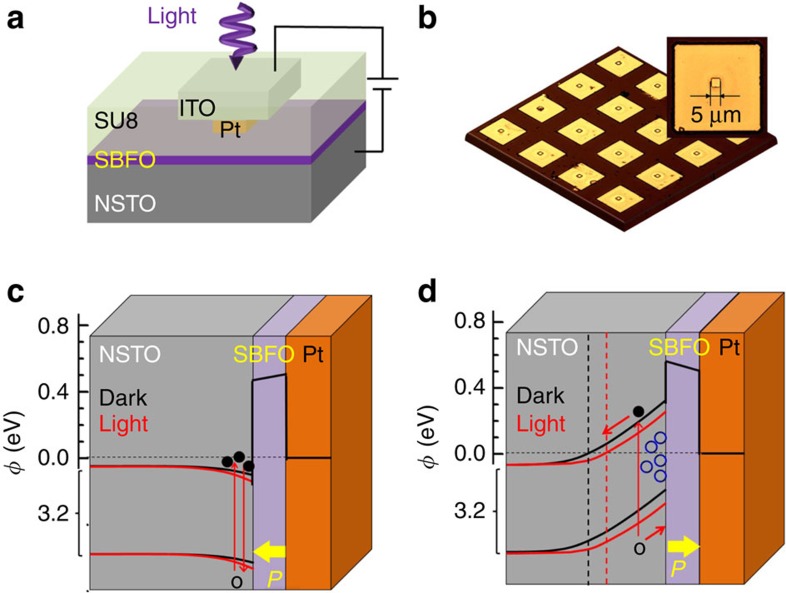
Mechanism of complementary electroresistance and photovoltaic effects. (**a**) Schematic representation of structure of the NSTO/SBFO/Pt FTJs. (**b**) Optical image of the as-prepared FTJs with an effective junction area of 5 μm × 5 μm (shown in the inset). Band alignment in the dark and under light illumination are shown for ferroelectric polarization (yellow arrow) pointing to either (**c**) the semiconducting NSTO electrode or (**d**) the metal electrode Pt. Polarization-modified carrier accumulation/depletion at the NSTO/SBFO interface contributes to the complementary electroresistance and PV effects. Electric-filed-induced drift of positively charged oxygen vacancies (blue circles) from the NSTO bulk towards the interface is also illustrated in **d**. Black filled (empty) circles represent photon-excited electrons (holes).

Schematics of the FTJ and the corresponding energy band diagrams are shown in Fig. 1, illustrating the complimentary effects of light illumination and electric field on the device transport. The device has a pushpin-type structure (Fig. 1a) with an effective junction area of 5 μm × 5 μm (Fig. 1b) (see Methods for details of device fabrication). The unique feature of the ITO/semi-transparent Pt top electrode allows light penetration, which enables the modulation of the junction transport by light illumination in addition to the polarization states as shown by the schematic band alignments in Fig. 1c,d. When the polarization is pointing to the left (right), NSTO interface is accumulated (depleted) due to the charge screening effect, which yields substantial changes of the barrier width in addition to the barrier height and hence leads to a giant TER effect. More importantly, the light illumination could change the band bending of the NSTO interface by exciting photo carriers, leading to additional tuning of the TER effect. Simultaneously, the isolation of excited electrons and holes due to the band bending of NSTO results in a PV effect, providing us a new route to read the non-volatile electronic state of FTJs.

### Characterization and ferroelectricity of SBFO films

**Figure 2:**
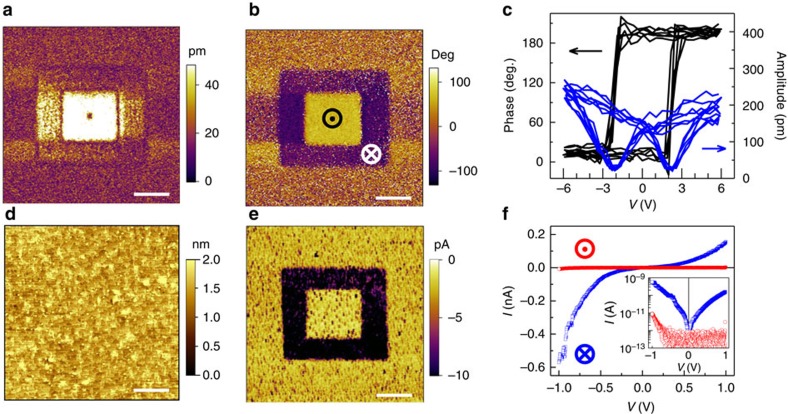
PFM and CAFM measurements on the ultrathin (3 nm) SBFO film. (**a**) PFM amplitude image and (**b**) phase image of square domains with opposite polarization directions written using an AFM tip with ±4 V bias. (**c**) Local PFM hysteresis curves. (**d**) AFM image of the film surface. The RMS roughness is 0.4 nm. (**e**) Current mapping taken at a low bias of −0.5 V after writing opposite domain patterns on the same film. (**f**) Local *I*−*V* curves for two opposite polarization domains (blue, down; red, up) by CAFM. Inset, *I*−*V* data on a log scale. Note that DC voltages were applied on the sample for current mapping and *I*−*V* curve measurements. Scale bars, 1 μm.

We first characterized the ferroelectricity and the polarization controlled resistive switching of the bare ferroelectric film. Epitaxial SBFO films of 3−9 nm were grown using pulsed-laser deposition (PLD) on (001) 0.7 wt.% NSTO single crystal substrates. Atomic force microscopy measurement on the 3 nm bare film shows smooth surface with roughness of 0.4 nm (shown in Fig. 2d). SBFO was chosen as the barrier due to its smaller leakage current compared with BFO (Supplementary Fig. 1). NSTO is a degenerate semiconductor and is used as both the growth substrate and the bottom electrode (see Methods for details). The basic structural properties of the films are presented in Supplementary Fig. 2.

The SBFO films were in-plane compressively strained, ensuring stabilization of ferroelectricity in the ultrathin films at room temperature, which was confirmed using piezoresponse force microscopy (PFM). As shown in Fig. 2a,b, clear amplitude and a phase contrast of ∼180^°^ were observed in the bare 3 nm SBFO film after writing square patterns with alternative +4 and −4 V biases applied on the PFM tip. These results clearly indicate the ferroelectric nature of the 3 nm SBFO film, which was further supported by the butterfly-like amplitude and hysteresis behaviour of the phase signal in the local PFM measurement, as shown in Fig. 2c. Similar ferroelectric properties were also observed in 6 and 9 nm films (Supplementary Figs 3,4). The acquired coercive electric field of ∼730 MV m^−1^ for 3 nm SBFO film is comparable with those reported for 5 nm BFO films (∼200−600 MV m^−1^) (ref. 25), whereas they are one or two orders of magnitude larger than those of thick BFO films (∼10 MV m^−1^) (ref. 39) and BFO single crystals (∼2 MV m^−1^) (ref. 5), indicating the important role of the interface in the switching behaviour of ultrathin ferroelectric films.

In conjunction with the PFM, we also investigated the local current switching properties of the as-grown SBFO film using conductive atomic force microscopy (CAFM). Current mapping was acquired by scanning a polarization-patterned area with a dc bias of −0.5 V applied on the sample (Fig. 2e). The current contrast clearly suggests that the polarization reversal modifies the local conduction of the 3 nm SBFO film. A larger current was observed for the downward-polarized domain, which is qualitatively consistent with the band alignment shown in Fig. 1c,d. *I−V* curves shown in Fig. 2f were acquired in local areas with opposite polarizations. The *I*−*V* curve of low-resistance state (LRS) shows a parabolic behaviour, consistent with the direct tunnelling across the ultrathin SBFO layer, whereas the *I*−*V* curve of high-resistance state (HRS) shows as an asymmetric Schottky-emission-type behaviour. A HR/LR ratio of up to ∼1,000 (inset of Fig. 2f) was obtained. Current mapping (Supplementary Figs 3b,e) and local *I*−*V* curves (Supplementary Fig. 5) were also performed on SBFO films with thicknesses of 6 and 9 nm. Different from the 3 nm ultrathin film, both HRS and LRS of thicker films showed typical Schottky-emission behaviour, indicating that the conduction of heterostructures with thick SBFO barriers is no longer governed by direct tunnelling.

### Transport mechanisms and voltage/light-induced TER of FTJs

**Figure 3:**
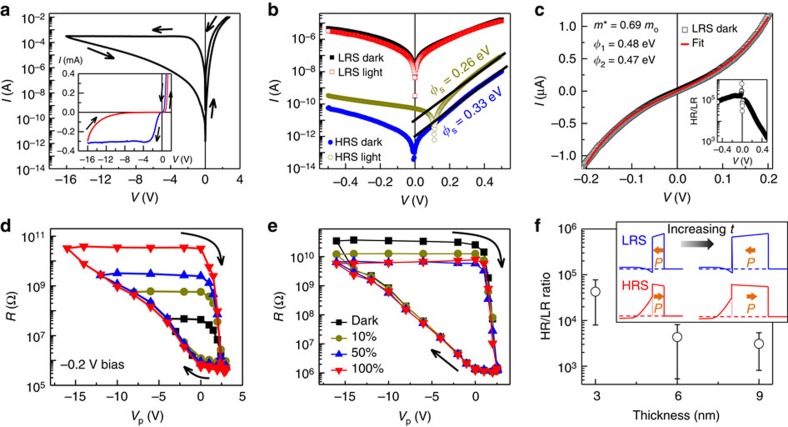
Tunable electroresistance controlled by voltage and light in NSTO/SBFO (3 nm)/Pt FTJs. (**a**) *I*−*V* switching characteristics of the FTJ as the voltage sweeps from 0 to +3 V, to −16 V and then back to 0 V. Inset is the same set of data shown on a linear scale. (**b**) Typical *I*−*V* data of LRS and HRS in the low-bias region after writing with voltages of +3 and −16 V, both in the dark and under UV light illumination. (**c**) Tunnelling behaviour of the LRS. Inset, HR/LR ratio in the dark calculated from the *I*−*V* curves shown in **b**. *R*−*V* hysteresis loops measured with (**d**) increasing voltage pulses in the dark and (**e**) increasing light intensities. (**f**) HR/LR ratio as a function of the SBFO thickness. The error bars represent the s.d. measured in 18 junctions for each barrier thickness. Inset is the schematics of corresponding band alignment.

Now we turn to the electric properties of FTJs with the ultrathin SBFO films being sandwiched between NSTO and Pt electrodes (Fig. 1a). For electric characterizations, applied voltage is defined as positive when the top Pt electrode is positively biased. Indium was soldered on the NSTO substrate to form good Ohmic contacts. Figure 3a shows the *I*−*V* characteristics of a typical junction by sweeping the voltage from 0 to 3 V, then to −16 V, and finally back to 0 V. Clear resistive switching was observed with the positive (negative) voltage leading to LRS (HRS). The rectification ratio at 3 V is 5.6 × 10^4^ for HRS, whereas it decreases to 32 for LRS. The prototypical diode behaviour observed in the high bias regime indicates that the Schottky barrier, rather than the tunnel barrier, dominates the resistance of the junction (Supplementary Fig. 6).

Figure 3b presents the *I*−*V* curves of the FTJ in the low-bias regime (−0.5 to +0.5 V) of LRS and HRS after applying voltage pulses of +3 and −16 V both in the dark and under UV light illumination. It was found that the nonlinear LRS *I*−*V* curve can be well described by the direct tunnelling theory based on the WKB approximation^19^,


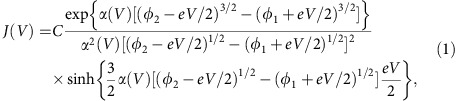


Where *C*=−(4*em*)/(9*π*^2^*ℏ*^3^) and *α*(*V*)=[4*d*(2*m*)^1/2^]/[3*ℏ*(*φ*_1_−*φ*_2_+*eV*)]. In this equations, *φ*_1,2_ are the tunnelling barrier heights at the two interfaces and *m* is the effective mass of electrons in the SBFO tunnel barrier. Figure 3c shows the fitting to the LRS curve using the following parameters: *φ*_1,2_=0.48 (0.47) eV for the NSTO/SBFO (Pt/SBFO) barrier and *m* of 0.69*m*_o_. Similar fitting results were achieved on nearly 20 junctions (Supplementary Fig. 7).

In contrast, for the *I*−*V* data of HRS, no reasonable fit to the direct tunnelling mechanism could be obtained (Supplementary Fig. 8). However, as shown in Fig. 3b, the conduction behaviour of HRS can be described by Schottky thermionic emission, for which the forward current is governed by the following equation^40^:


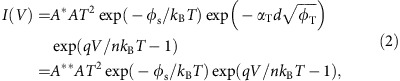


where *A* is the junction area, *A* is the standard Richardson constant (600 A cm^−2^ K^−2^ by assuming an effective mass of 5*m*_0_ for NSTO),^41^ *φ*_s_ is the Schottky barrier height, *T* is the temperature and *n* is the ideality factor. The term 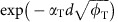 denotes the current-reducing effect due to the tunnelling barrier, where *α*_T_=2(2*qm*)^1/2^/*ℏ*, *d* is the thickness of SBFO and *φ*_T_ is the average tunnelling barrier height. The impact of the tunnelling barrier is reflected by the reduced Richardson constant *A*(∼0.2 A m^−2^ K^−2^) by assuming a *φ*_T_ of 0.475 eV for simplicity, the same as that of LRS). The Schottky barrier height *φ*_s_ of HRS can then be estimated from fitting the forward current to equation 2, which provides 0.33 and 0.26 eV for *φ*_s_ in the dark and under illumination, respectively. Furthermore, the depletion layer thickness *W*_d_ could be estimated using the following relationship: *φ*_s_=(*qN*_d_/2*ɛ*_0_*ɛ*_s_)*W*_d_^2^ (ref. 40), where *N*_d_ (∼10^20^ cm^−3^) and *ɛ*_s_ (∼200) are the free carrier density and the static dielectric constant of NSTO^22^, respectively. This estimation provides a *W*_d_ of∼8.5 nm for the FTJ in the dark, which decreases to∼7.6 nm under light illumination. In addition, note that the derived ideality factor *n* of 1.9 deviates from 1, which is consistent with the simultaneous existence of tunnelling electrons^40^,^42^. The direct tunnelling character of LRS and the contribution of electron tunnelling to the Schottky thermionic emission of HRS were further confirmed by temperature-dependent *I*−*V* measurements (Supplementary Fig. 9 and Supplementary Table 1).

Based on the above analysis, we conclude that in the low-bias regime (<0.2 V), the LRS is dominated by the direct tunnelling, whereas the HRS is dominated by the Schottky thermionic emission. This conclusion is consistent with the band structure of the Pt/SBFO/NSTO tunnel junction shown in Fig. 1c. In the LRS, the polarization of SBFO points to the NSTO electrode, which leads to the accumulation of charges at the NSTO/SBFO interface, and electrons can directly tunnel through the ultrathin SBFO barrier. In contrast, in the HRS, the polarization of SBFO points away from NSTO, the positive screening charges lead to upward bending of the NSTO conducting band, and the Schottky barrier is thus significantly enhanced. This increase in the depletion region in NSTO results in the onset of HRS, which is accompanied by a change in the transport mechanism from direct tunnelling to Schottky emission.

Figure 3d presents typical non-volatile resistive memory loops controlled by voltage pulses. The junction was first set to LRS by a pulse voltage of +3 V, followed by sweeping the voltage to −4 (or −8, −12 and −16) V and back to +3 V. The resistance of FTJ was read at a bias of −0.2 V. A series of intermediate resistance states can be achieved by adjusting the magnitude of the applied maximum negative voltage; thus, the device behaves as a memristor^43^,^44^. A giant HR/LR ratio of approximately 10^5^ was finally achieved, which is consistent with that calculated directly from the *I*−*V* curves, as shown in the inset of Fig. 3c. This colossal TER is considerably larger than that previously reported for a BFO-based FTJ (∼0.8 at 80 K) (ref. 45) and 10-times larger than FTJs based on tetragonal-BFO^28^. To the best of our knowledge, the TER achieved here is the highest ever reported for FTJs^36^. Furthermore, this colossal TER exhibits only slight decrease on increasing negative bias (see the inset of Fig. 3c), allowing to increase the readout current levels without losing the performance.

Interestingly, the *R*−*V* loops in Fig. 3d are highly asymmetric, that is, the transition from HRS to LRS is abrupt, whereas the transition from LRS to HRS is gradual. This is different from the symmetric ferroelectric hysteresis loops (Fig. 2c), where full polarization-reversal occurs at approximately 2–3 V and no strong imprint effect was observed. Thus, we believe that in addition to the polarization reversal, other factors such as drifting of oxygen vacancies under the intense electric field could contribute to the observed giant ER effect^29^,^46^. As shown in Fig. 1d, the accumulation of oxygen vacancies at the NSTO/SBFO interface under the negative electric field enhances the depletion layer and consequently increases the electroresistance. In addition, we found that the ER magnitude decreases with increasing SBFO thickness (see Fig. 3f and Supplementary Fig. 10), which is consistent with the fact that the effect of tuning NSTO barrier on ER becomes weaker when the thicker SBFO barrier dominates the junction transport (inset of Fig. 3f). Because of the same reason, in FTJs with thicker SBFO (6 and 9 nm), Schottky diode effect, instead of electron tunnelling, dominates the transport behaviour of both LRS and HRS, which is similar to the observations made on thick BFO films^47^.

More interestingly, we found that the electroresistance of FTJs could also be modulated by UV-light illumination. As shown in Fig. 3b, UV-light illumination produced almost no effect on LRS, but notable tuning was observed for HRS. The modulation of electroresistance by illumination was also confirmed by the resistance memory loops (Fig. 3e) measured under illumination with different light intensities. Furthermore, switch cycling tests were conducted under both dark and light conditions (Supplementary Fig. 11). In general, the HR/LR ratio was reduced by∼10-fold when the FTJs were exposed to UV light. This result is consistent with the Schottky barrier reduction of ∼0.07 eV obtained from fitting the HRS *I*−*V* curves. Because this effect was observed only for HRS and because NSTO is the main light absorber in the devices, we believe that this effect is correlated with the tuning of the NSTO depletion layer near the SBFO/NSTO interface. The light excited free electrons will diffuse towards the NSTO interface and reduce the depletion width and barrier height for HRS (Fig. 1d). This was further confirmed by the light-tuning effect observed in capacitance measurements (Supplementary Fig. 12). Such complementary modulations of the resistance states by electric field and light illumination produce additional resistance states and enhance the memory density of the FTJs.

### PV effect and non-destructive reading of the memory states

For a metal−insulator−semiconductor heterostructure, when the illuminated wavelength matches the optical bandgap of the active material, photo-excitation of charge carriers occurs. The generated electrons and holes will be separated by the internal electric field, which leads to the PV effect^40^. In our case, the main light-absorbing layer is NSTO rather than SBFO because of the considerably thinner character of the latter, which was confirmed by investigating the wavelength dependence of the PV effect (Supplementary Fig. 13). In other words, the short-circuit current *I*_sc_ (current at zero bias) and open-circuit voltage *V*_oc_ (voltage at zero current) are mainly determined by the depletion layer near the NSTO/SBFO interface. Consequently, the PV effect is expected to be dependent on the memory states of the FTJ and thus tunable.

**Figure 4:**
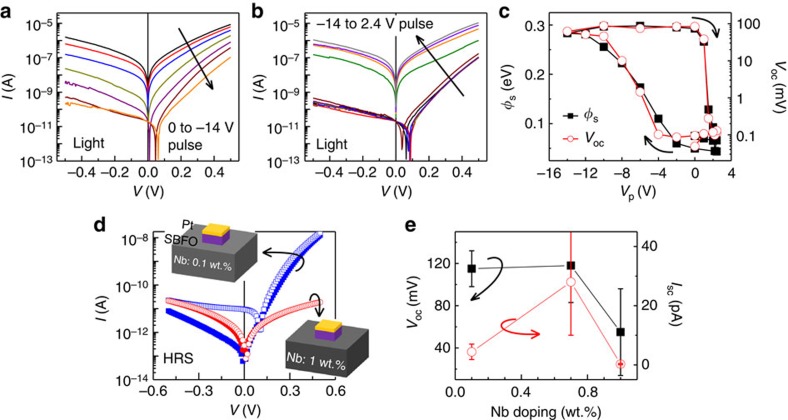
Electrically tuned PV effect in NSTO/SBFO/Pt FTJs. Typical *I*−*V* curves of FTJ measured under light illumination (**a**,**b**). The successive voltage pulses are 0, −2, −4, −6, −8, −10 and −14 V in **a**; −14, −10, −6, −2, 0, 1, 1.5, 2, 2.2 and 2.4 V in **b**. The device was first set to LRS by a +2.4 V pulse before the measurement. (**c**) Schottky barrier *φ*_s_ (left) and *V*_oc_ (right) as a function of the voltage pulse. *φ*_s_ is obtained from the thermionic emission fitting to the forward *I*−*V* curves measured in the dark. (**d**) Typical HRS *I*−*V* curves measured on FTJs with Nb doping levels of 0.1 and 1 wt.% (solid, dark; empty, light). (**e**) *V*_oc_ and *I*_sc_ of HRS measured on FTJs using NSTO substrate with different Nb doping levels. The error bars represent the s.d. measured in 18 junctions for each doping level.

Figure 4a-4b show the *I*−*V* curves measured under light illumination after switching with a series of voltage pulses (*V*_p_) from 0 to −14 V (LRS to HRS) and from −14 to 2.4 V (HRS to LRS), respectively. We found that the HRS *I*−*V* curves exhibit notable shifts along the voltage axis, and *V*_oc_ is as high as ∼100 mV. In contrast, *V*_oc_ is negligible small for LRS (<0.1 mV). Whereas no shift has been observed for *I*−*V* curves measured in the dark (Supplementary Fig. 14). This result unambiguously suggests that the magnitude of *V*_oc_ depends on the band bending in NSTO near the NSTO/BFO interface (Fig. 1c,d). The strong upward band bending in the HRS (Fig. 1d) drives the photo-generated electrons into the NSTO bulk, and the holes tunnel through the SBFO barrier, leading to the PV effect. Both the resistance state and the PV effect exhibit similar dependence on the switching voltage bias, indicating the shared mechanism related to the depletion/accumulation of NSTO near the interface, revealing the importance of the electric field induced polarization reversal and the interface charge redistribution (Fig. 1c,d). Consequently, the evolution of *V*_oc_ as a function of the applied voltage pulses (Fig. 4c, right) is very similar to that of the Schottky barrier (Fig. 4c left, obtained from the fitting of *I*−*V* curves in the dark) and the resistance memory loops (Fig. 3e). Furthermore, the dependence of *I*_sc_ on the applied voltage pulses also shows hysteresis behaviour (Supplementary Fig. 15). In general, such a resistance-state-dependent PV effect provides a new route by using *V*_oc_ (or *I*_sc_) for sensing the memory states of FTJs in addition to the conventional resistive reading. This new route is non-destructive and reliable because light illumination will not change the polarization state of the devices. Because *V*_oc_ is in principle independent of the lateral size of the junctions, this *V*_oc_-based reading scheme is amenable to the miniaturization of data storage devices and promising to facilitate the high-density integration.

Note that the PV effect observed in our metal/ferroelectric/metal FTJs is different from that recently reported for FE capacitors in which the ferroelectric layer itself is the active light-sensing component^31^. The present device scheme allows us to engineer the PV effect by adjusting the Nb doping level of the NSTO substrates. Figure 4d shows the typical HRS *I*−*V* curves of FTJs fabricated on NSTO substrates with Nb doping levels of 0.1 and 1 wt.%. Although the NSTO substrate with 1 wt.% Nb doping has the strongest light absorption (Supplementary Fig. 16), its higher carrier concentration leads to thinner depletion layer and weaker PV effect^40^. In addition, different with the highly asymmetric HRS *I*−*V* curves of FTJs based on NSTO (0.1 and 0.7 wt.%), the HRS *I*−*V* curves of FTJ based on NSTO (1 wt.%) show parabolic direct tunnelling behaviour, in line with the much reduced Schottky barrier formed at the NSTO (1 wt.%)/SBFO interface. The dependence of the PV effect on the Nb doping level is summarized in Fig. 4e. Clearly, FTJs fabricated on 0.7 wt.% doped NSTO substrate presents the best PV performance.

### Device performance and reliability

**Figure 5:**
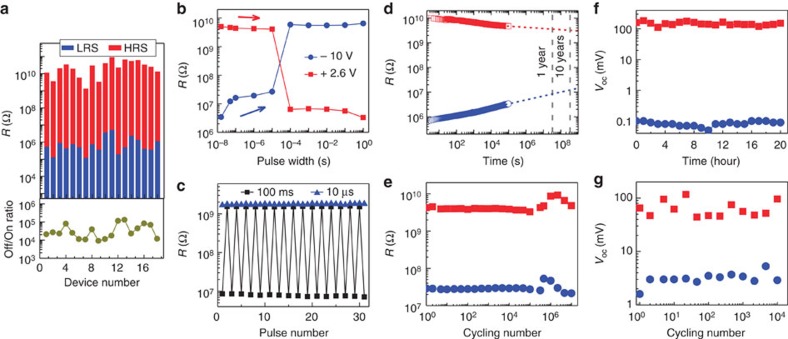
Retention and fatigue properties of NSTO/SBFO (3 nm)/Pt FTJs. (**a**) Off and on resistance states read at a −0.2 V bias and the corresponding off/on ratios measured on 18 junctions. (**b**) Resistance evolution of the FTJ after writing with increasing voltage pulse widths from 16 ns to 1 s. Writing voltages of −10 and +2.6 V were used. (**c**) Consecutive resistance switching cycles induced by voltage pulses with durations of 10 μs and 100 ms. (**d**) Retention properties of the HRS and the LRS after setting by −16 and +3 V with pulse width of 1 s. (**e**) Fatigue characteristics up to 10^7^ cycles of a typical FTJ device. Each cycle consists of writing at +2.6 V, reading at −0.2 V, writing at −10 V and reading at −0.2 V. The pulse width is 10 ms. (**f**) Retention and (**g**) fatigue behaviours of *V*_oc_ of typical devices.

For practical non-volatile memory applications, a large off/on resistance ratio, fast writing speed, long data retention and stable fatigue properties are the most important figures of merit. Fig. 5a shows the HRS and LRS reading at a −0.2 V bias for 18 randomly selected devices with the SBFO barrier thickness of 3 nm. The resistance ratios fall in the range of 10^4^–10^5^, indicating the high yield and good uniformity of the FTJs. Note that the writing current density of our FTJs is only ∼2 × 10^4^ A cm^−2^, which is comparable with the previously reported FTJ based on tetragonal BFO thin films^11^, and two to three orders smaller than those of spin-based devices such as magnetic random access memories (MRAMs)^48^, spin-transfer-torque-based MRAM^33^, and spin–orbit-torque-based MRAM^49^.

To investigate the operation speed of resistive switching, we measured the influence of the poling pulse width on the resistance switching from HRS (LRS) to LRS (HRS). Before the measurements, the device was first set to HRS (LRS) by applying a voltage pulse of −10 V (+2.6 V) with duration of 1 s. Then, square pulses with varied pulse widths of 16 ns to 1 s were applied on the device, and the resistance of FTJ was subsequently recorded at a bias of −0.2 V. As shown in Fig. 5b, resistance switching emerges when the pulse width exceeds ∼10 μs. This result was also confirmed by the characteristics of the resistive switching data measured using different pulse widths, as shown in Fig. 5c. The switching of the FTJs is faster than that of flash memory^50^ but slower than resistive random access memory^51^ and MRAM^52^. A writing speed of up to 10 ns has recently been realized in LSMO/BTO/Co FTJs with sizes on the nanometre scale^13^,^27^, suggesting that our FTJs still have the potential for improvement.

Generally speaking, there are two factors that limit the writing speed of FTJs: the switching time of the polarization and the field-induced drift of charged species at the interface. Considering the switching of the polarization could be as fast as 1 ns for ferroelectrics^31^, we believe that the switching speed of our FTJs is mainly limited by the dynamics of the drifting charges. Semiconducting NSTO has a depletion layer thickness of ∼8 nm, which is much larger than that of LSMO (∼0.2–1.9 nm) (ref. 16) and may lead to a longer switching time. Since the writing speed determines the ultimate operating bandwidth of integrated FTJ memories, more in-depth studies are warranted in future.

Figure 5d shows the resistance retention at a reading bias of −0.2 V. The values of HRS and LRS slightly decrease and increase with time, respectively, but an off/on ratio of more than 10^3^ is still retained after 10^5^ s. This value is ∼260 after 10 years through a simple extrapolation, suggesting the excellent non-volatility of our device^22^,^53^. Furthermore, the off and on states are stable under repeated bipolar switching cycles, as shown in Fig. 5e. Voltage pulses of −10 and +2.6 V with a pulse width of 10 ms were applied in this endurance test. Slight resistance decreases were observed for both states with increasing cycle numbers, and a large off/on ratio of ∼200 was preserved after∼10^7^ writing cycles. This fatigue property of our FTJs is comparable or better than those in previous reports, for example on BTO based FTJs (off/on ratio of ∼100 over 3,000 writing cycles)^22^, and BFO-based FTJs (off/on ratio of ∼100 up to 4 × 10^6^ cycles)^53^. In addition, the HR and LR states of our FTJs appear to be more stable without much resistive fluctuation compared with the previously reported FTJs based on BFO barrier^53^. The substantial improvements in both the fatigue and the stability of the non-volatile states could be attributed to the Sm doping in the ultrathin ferroelectric BFO layers. As previously reported, rare earth doping could reduce the migration of oxygen vacancies^54^, which has been widely believed to be one of the microscopic origins of fatigue in ferroelectric oxides^55^. We also monitored the retention and fatigue behaviours of *V*_oc_ for HRS and LRS under light illumination (Fig. 5f,g), no deterioration in retention up to 20 h and in fatigue up to 10^4^ cycles was observed, suggesting the feasibility and robustness of the PV route for reading the non-volatile states of FTJs.

## Discussion

In this work, we demonstrated that using an ultrathin (3 nm) SBFO as the tunnel barrier, in conjunction with a semiconducting Nb:STO as the bottom electrode, can significantly improve the performance of FTJs. In particular, the off/on ratio reaches a giant value of 10^5^, which, to the best of knowledge, is the highest reported to date in the literature. One important factor underlying the achieved improvements is the excellent ferroelectric properties and the ultra-low leakage current of the 3 nm SBFO layer. BFO and its doped variants are known for their high spontaneous polarization; for example, the polarization of bulk BFO reaches ∼70 μC cm^−2^ along [001] (ref. 39), whereas it is ∼26 μC cm^−2^ for BTO^56^. Ultralow leakage current also plays a very important role in increasing the performance of FTJs because the undesired leakage current would suppress the switching effect of ferroelectric polarization and reduce the off/on ratio of FTJs. Doping Sm to a level of 10% was found to significantly reduce the leakage current without losing the polarization (Supplementary Fig. 1). In addition, the piezoelectric coefficient could be significantly enhanced after Sm doping^57^. All these factors are potentially beneficial to achieving TER with even larger magnitudes^7^,^12^. Overall, our results highlight ultrathin SBFO layers as an excellent choice as the tunnel barrier in high-performance FTJs.

The semiconducting NSTO electrode is another important factor behind the excellent performance of our FTJs. In previous reports, the memristive behaviour observed in FTJs such as LSMO/BTO/Co was attributed to the electric modulation of ferroelectric domains^27^ or the interface-charge redistribution^29^. In the present work, we demonstrated that the colossal TER originates from the bias-induced transition between the direct tunnelling across the SBFO barrier and the thermionic emission over the Schottky barrier at the NSTO/SBFO interface. We should note that strictly speaking, our Pt/SBFO/NSTO heterostructures are no longer FTJs in the HRS because the junction transport is predominantly limited by the Schottky barrier instead of the tunnel barrier. This is intrinsically different from the transport mechanism reported previously for most other FTJs^13^,^14^,^16^,^18^,^19^,^20^,^21^,^58^, where the direct tunnelling was claimed to be responsible for both HRS and LRS. There have been only a few reports on the thermionic emission effect in FTJs^36^,^59^. In fact, our findings are qualitatively consistent with the results from Pantel and Alexe^60^. Their calculations showed that switching between different transport mechanisms by polarization reversal could yield large electroresistance. In our case, the effective barrier widths were estimated as ∼3 and∼11.5 nm for LRS and HRS, respectively. Such a large modulation in the barrier width naturally leads to the transition of transport mechanism between direct tunnelling and Schottky emission, which is the main mechanism underlying the colossal TER achieved in our FTJs.

Another benefit of using such a semiconducting NSTO electrode is the light-enabled tunability observed in the FTJ transport. On the one hand, light illumination can be used as an additional parameter to control the resistance state of the FTJs (Fig. 3e and Supplementary Fig. 11), which allows the realization of multiple non-volatile states and enhances the data storage capability. On the other hand, the observed PV effect provides an alternative route for sensing the electronic states of FTJs non-destructively.

In summary, by doping the ultrathin ferroelectric layer and using the light-absorbing semiconducting substrate, we realized a colossal TER of up to 10^5^ in NSTO/SBFO/Pt FTJs, which, to the best of our knowledge, is the largest ever reported in such devices. We demonstrated the complementary modulation of the TER by light illumination and electric field, as well as the PV effect for the first time in FTJs. The results reported here establish a strong connection between the giant TER/PV effects and the modulation of the NSTO interface triggered by applied electric field and light illumination. The good device performance of these FTJs in terms of retention, endurance, and reproducibility underscores their potential for future device applications.

## Methods

### Device preparation

The PLD technique was used to prepare epitaxial Sm_0.1_Bi_0.9_FeO_3_ films on a (001) 0.7 wt% Nb-doped SrTiO_3_ substrate. Similar to our previous reports on growing oxide heterostructures, KrF excimer laser (248 nm) was used with an energy density of 1 J cm^−2^ and a repetition rate of 3 Hz. Films with thicknesses from 3 to 9 nm were grown using a Sm_0.1_Bi_0.9_FeO_3_ target at a substrate temperature of 600 °C and an oxygen pressure of 10 mTorr. Pushpin-shaped FTJs with top Pt (15 nm)/ITO (350 nm) electrodes were then prepared on the films using a two-step photolithography technique, and the FTJs had a working size of 5 μm × 5 μm. In detail, the sample was first spin coated with negative photoresist SU8-2000.5 (MicroChem) at 3,500 r.p.m. for 30 s and baked at 100 °C for 60 s. The sample was then attached to a photomask for UV light exposure with an intensity of 70 mJ cm^−2^, and the patterns were obtained by developing in SU8 developer (MicroChem) for 2 min. The top openings (50 μm × 50 μm) were then fabricated by spin coating positive photoresist AZ1505 (MicroChemicals) at 3,000 r.p.m., exposed under UV light of 20 mJ cm^−2^ and followed by developing in AZ726 (MicroChemicals) for 20 s. After photolithography, Pt/ITO top electrodes were prepared by magnetic sputtering and lift-off.

### Electrical characterizations

PFM and conducting atomic force microscopy measurements were performed using a commercial atomic force microscope (Asylum Research MFP-3D) with Pt/Ir-coated Si cantilever tips and diamond-coated Si cantilever tips, respectively. In typical PFM measurements, an ac voltage of amplitude of 600 mV and at a contact resonance frequency of ∼300 kHz was applied on the tip. For the local electric measurements, the bias voltage was applied on the sample. *I*−*V* measurements on FTJs were performed on a probe station (Cascade MPS150) equipped with a multi-SourceMeter (Keithley 2635 A). UV light was provided by a halogen lamp (Asahi Max-303) with wavelength from 250–385 nm and illumination energy density of up to 60 mW cm^−2^. Voltage pulses were supplied by an arbitrary waveform generator (Agilent 33522 A). For the fatigue measurements, the voltage stress pulses were applied using a Multiferroic tester (Radiant Technologies). Unless specified otherwise, the setting/resetting voltage pulses have a fixed length of 1 s. Capacitance was measured using a LCR meter (Agilent E4980A) with a frequency ranging from 20 Hz to 20 MHz. In all measurements, the bottom electrodes were grounded and voltages were applied on the top electrodes.

### Optical characterizations

To check the absorption of Nb doped SrTiO_3_, 100 nm thin films of different doping levels were grown by PLD on double-side-polished quartz substrate at a temperature of 750 °C and in vacuum (∼2 × 10^−7^ torr) followed by annealing at 600 °C for 30 min under oxygen pressure of 10 mtorr. Then the transmittance was characterized by photospectrometry (Cary 5000, Agilent technologies) using quartz substrate as the reference.

## Additional information

**How to cite this article:** Hu, W. J. *et al.* Optically controlled electroresistance and electrically controlled photovoltage in ferroelectric tunnel junctions. *Nat. Commun.* 7:10808 doi: 10.1038/ncomms10808 (2016).

## Supplementary Material

Supplementary Information Supplementary Figures 1-16, Supplementary Table 1 and Supplementary References
