# Supplementary material for: Optically controlled electroresistance and electrically controlled photovoltage in ferroelectric tunnel junctions
Source: Nat Commun. 2016 Feb 29;7:10808. doi: 10.1038/ncomms10808 (PMC4773477; doi:10.1038/ncomms10808)
Supplement: Supplementary Information — Supplementary Figures 1-16, Supplementary Table 1 and Supplementary References [file ncomms10808-s1.pdf]

Supplementary Figures

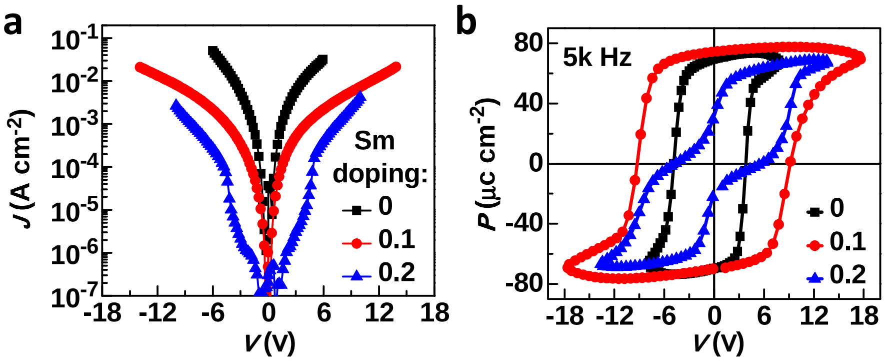

Supplementary Figure 1 | Electric properties of 200 nm $Sm_xBi_{1-x}FeO_3$ (x = 0, 0.1, 0.2) films. (a) The leakage current and (b) the ferroelectric hysteresis. As can be seen, the leakage current of $Sm_{0.1}Bi_{0.9}FeO_3$ (SBFO, 200 nm) is more than 10 times smaller than that of pure $BiFeO_3$(BFO). A further reducing of the leakage current by 3 orders in magnitude could be achieved by doping Sm to a level of 0.2, which however is accompanied with a strong depression of remanent polarization from 70 $\mu C\ cm^{-2}$ to 25 $\mu C\ cm^{-2}$ due to its paraelectric ground state.[1] Based on this investigation, we chose SBFO as the tunnel barrier in order to get the giant TER effect.

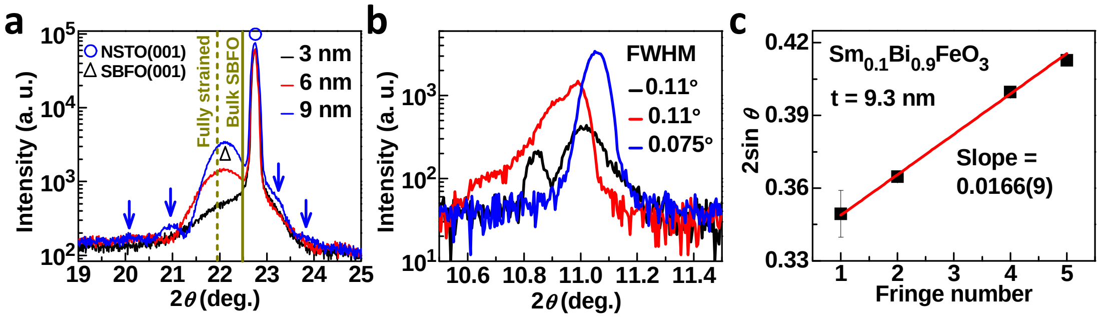

Supplementary Figure 2 | Basic structural characterization of $Sm_{0.1}Bi_{0.9}FeO_3$ films. (a) X-ray diffraction 2θ scans of the films with different nominal thickness of 3 nm, 6 nm, and 9 nm. No secondary phase was observed. (b) Rocking curves of the films with FWHM of less than 0.11^{o} suggest the good quality of the films. (c) Fringe position *vs.* fringe number for film with nominal thickness of 9 nm. The errors come from the standard deviation of the fringe peak position. The thickness determined

from the slope of this plot (~ 9.3 nm) is close to the nominal value (9 nm) determined from the thickness calibration through growing time dependence.

For these films, only the SBFO (00l) peak appears, indicating good epitaxy of the films with the Nb-doped $SrTiO_3$ (NSTO) substrate. SBFO (a_{bulk} ~ 3.952 Å, ref. 2) films were expected to subject an in-plane compressive strain from STO (a = 3.905 Å) due to the lattice mismatch (~ 1.2%). The predicted positions for fully strained SBFO and bulk SBFO (001) are shown as the dotted and the solid line, respectively, in Supplementary Fig. 2a. It is seen that 3 nm SBFO film is fully strained and some structural relaxation occurs with increasing the film thickness. This compressive in-plane strain is benefit for ensuring a high polarization for the ultrathin films.

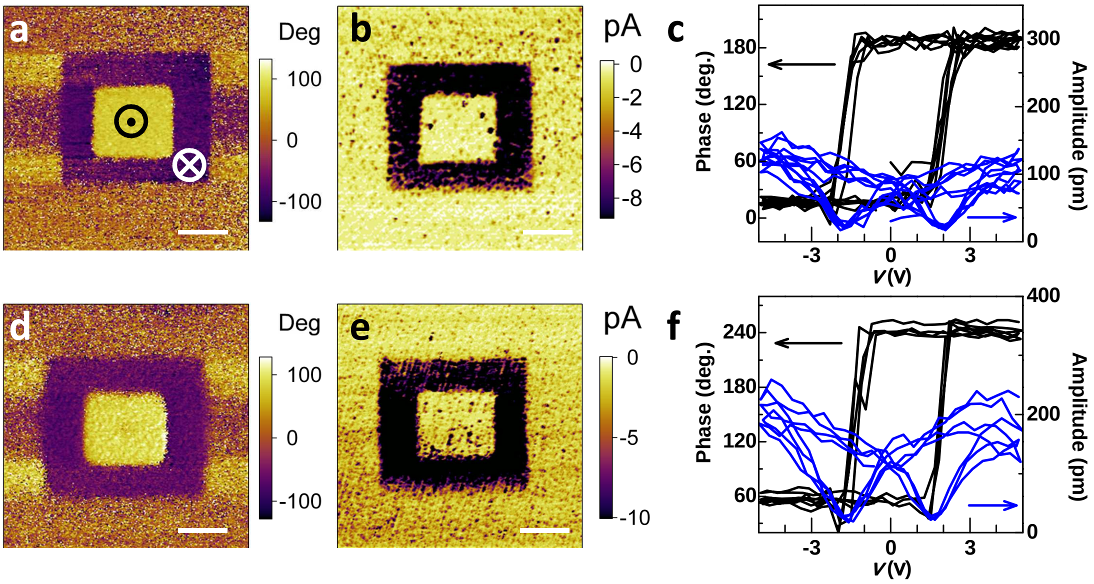

Supplementary Figure 3 | PFM and CAFM analyses of the SBFO films. The film thickness are 6 nm (top) and 9 nm (bottom), respectively. (a) (d) PFM phase image of domains with opposite polarizations. (b) (e) Corresponding CAFM mappings. The writing voltages are +4V and −4V, respectively. (c) (f) Local ferroelectric loops. Scale bars, 1 μm.

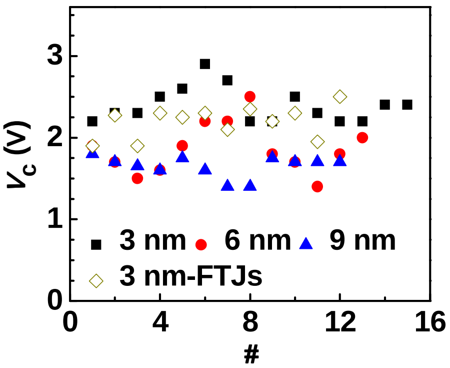

Supplementary Figure 4 | The statistics of coercivity. Generally, coercivity of the 3 nm bare SBFO film is larger than that of the 9 nm film, and coercivity of the bare film and of FTJs with top Pt electrodes are similar.

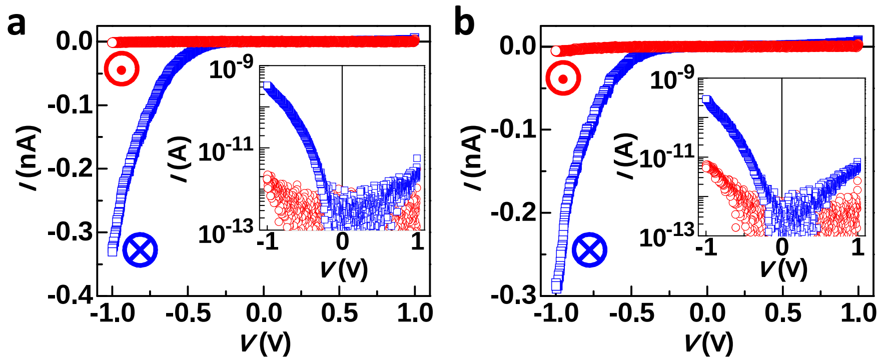

Supplementary Figure 5 | Polarization dependent *I*-*V* characteristics of SBFO films. The film thickness are (a) 6 nm and (b) 9 nm, respectively (Blue: with polarization pointing down; red: with polarization pointing up). Inset, the same data in the logarithmic scale.

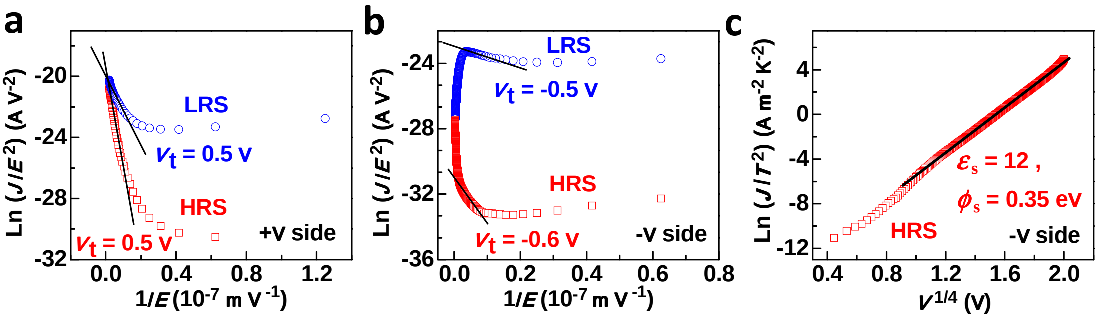

Supplementary Figure 6 | Typical *I*−*V* characteristics analysis of NSTO (0.7 wt. %)/SBFO (3 nm)/Pt FTJs in the high bias region (above 0.5 V). Fowler-Nordheim tunneling describes the data well for (a) +V side from ~ 0.5 V to ~

3 V, and (b) –V side from ~ −0.5 V to ~ −3 V. (c) Schottky thermionic emission well describes the conducting behaviour in the reverse direction for HRS.

While we hardly explored the conducting behaviour of the tunnel junction at high bias to avoid the dielectric breakdown, we expect that with large enough electric field, the band bending of the SBFO barrier will be strong enough and the electrons will tunnel through a triangular-shape barrier, which is described by the Fowler-Nordheim (FN) equation,[3]

$$J_{\mathrm{FN}} = \frac{q^2E^2}{16\pi^2\hbar\bar{\phi}}\exp[\frac{-4\sqrt{2m^*}(q\bar{\phi})^{3/2}}{3\hbar qE}], \quad (1)$$

The FN plots in Supplementary Figs. 6a and 6b qualitatively show that in the high bias region (~ 0.5 V to 3 V or −0.5 V to −3 V), both the LR and HR $I-V$ curves are indeed described by the FN tunneling. With the voltage continues increasing in the reverse direction, the depletion layer at the NSTO interface increases further. Thereby, the reverse-biased Schottky barrier rather than the tunnelling barrier dominates the conducting behaviour. The reverse current gradually increases with increasing the voltage bias due to the Schottky-barrier lowering effect. The equation of the current density is[3-5]

$$J_{\mathrm{s}} = A^{**}T^2\exp[-\frac{q}{k_{\mathrm{B}}T}(\phi_{\mathrm{s}} - (\frac{qE_{\mathrm{m}}}{4\pi\varepsilon_0\varepsilon_{\mathrm{op}}})^{1/2})], \quad (2)$$

where E_{m} is the maximum electric field at the SBFO/NSTO interface, $E_{\mathrm{m}} = \sqrt{\frac{2qN_{\mathrm{eff}}(V+V_{\mathrm{bi}}^{'})}{\varepsilon_0\varepsilon_{\mathrm{st}}}} \pm \frac{P}{\varepsilon_0\varepsilon_{\mathrm{st}}}$. ϕ_{s}, $\varepsilon_{\mathrm{op}}$, $\varepsilon_{\mathrm{st}}$, N_{eff} are the Schottky barrier, the optical dielectric constant, the static dielectric constant, and the effective charge density in the depletion region of NSTO, respectively. P is the polarization of the SBFO barrier. A^{**} is the effective Richardson constant (~ 0.2 A m^{-2} K^{-2}, see main text). For sufficient high electric field, the polarization term can be ignored and Supplementary Equation 2 could be simplified as:

$$Ln(J_{\mathrm{s}}/T^2) = LnA^{**} - \frac{q\phi_{\mathrm{s}}}{k_{\mathrm{B}}T} + \frac{q}{k_{\mathrm{B}}T}(\frac{q^3N_{\mathrm{D}}}{8\pi^2\varepsilon_0^3\varepsilon_{\mathrm{op}}^2\varepsilon_{\mathrm{st}}})^{1/4}V^{1/4}, \quad (3)$$

As shown in Supplementary Fig. 6c, Ln J/T^2 *vs.* $V^{1/4}$ for HRS shows as a straight line above ~ 2 V and can be well fitted by this equation. The obtained Schottky barrier ϕ_s is 0.35 eV by assuming $m^* = 5m_o$ for NSTO,[6] which is consistent with that obtained in the low bias region in the main text. The acquired dielectric constant $\varepsilon_s = (\varepsilon_{op}^2 \varepsilon_{st})^{1/3} =$ 12 for a 10^{20} cm^{-3} doping concentration. This value is reasonable if adopting $\varepsilon_{op} = 5$ and $\varepsilon_{st} = 200$ for NSTO,[7,8] which leads to $\varepsilon_s = 17$.

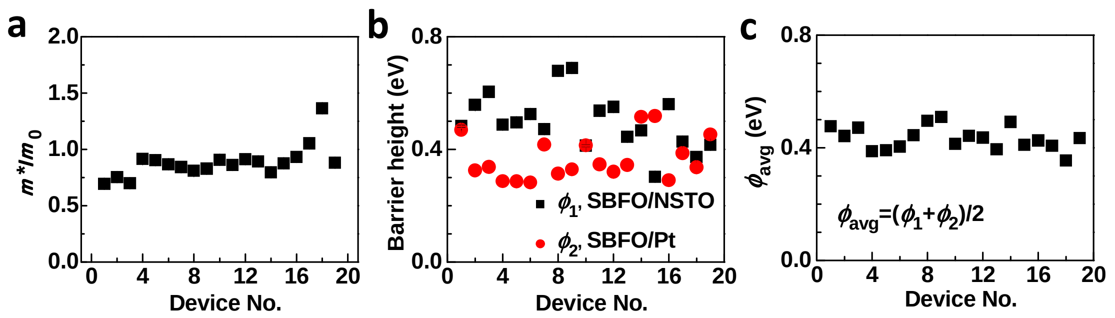

Supplementary Figure 7 | Fitting result of LRS by the trapezoidal barrier model. (a) The effective mass m^*, (b) the tunnel barrier height of SBFO/NSTO (ϕ_1) and SBFO/Pt (ϕ_2) interface, and (c) the average barrier height ϕ_{avg}. 19 devices [NSTO (0.7 wt. %)/SBFO (3 nm)/Pt] have been investigated.

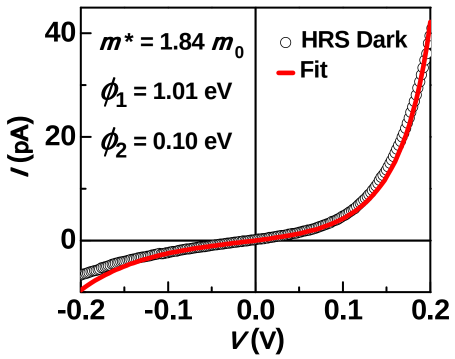

Supplementary Figure 8 | Direct tunneling fitting of the *I*−*V* curve for HRS. The direct tunneling fitting of the HRS *I*−*V* data for FTJ of NSTO (0.7 wt. %)/SBFO (3 nm)/Pt results in $\phi_{1,2} = 1.01$ (0.10) eV for NSTO/SBFO (SBFO/Pt) interface and m^* of 1.84 m_o. However, the fitting is not as good as the Schottky thermionic emission model shown below, especially for voltage bias beyond the range of −0.1 V to 0.1 V.

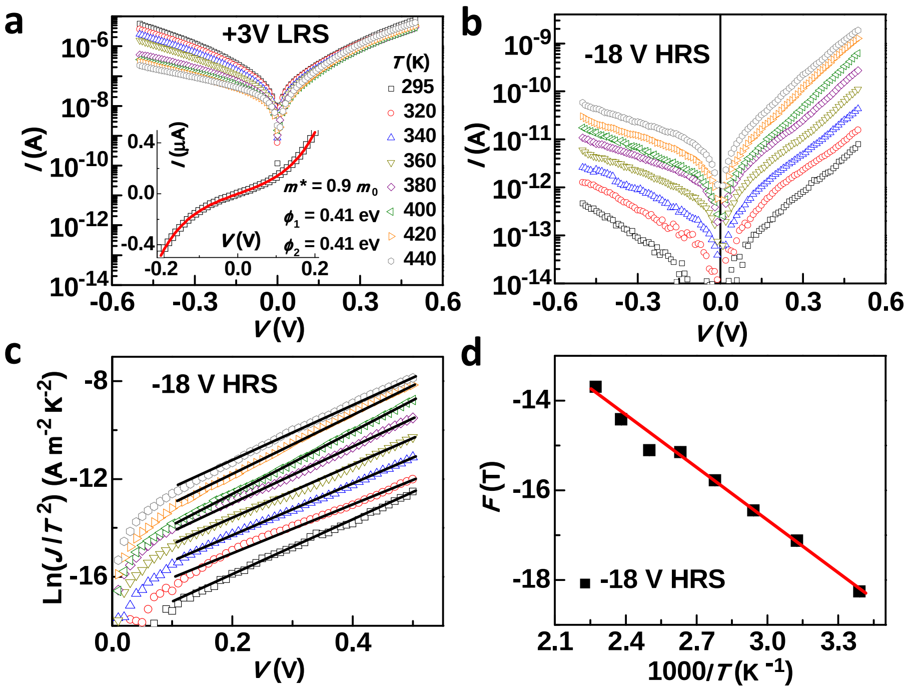

Supplementary Figure 9 | The thermal behaviour of LRS and HRS. *I*−*V* curves of (a) LRS and (b) HRS for NSTO (0.7 wt. %)/SBFO (3 nm)/Pt FTJ at different temperatures from 295 K to 440 K. LRS and HRS are obtained by +3 V and −18 V (with pulse width of 1 s) setting at room temperature, respectively. Inset of (a), the direct tunnelling fitting of LRS. (c) The presentation of HRS data in the forward direction in the form of Schottky thermionic emission. (d) *T*-dependent *F* (T) from which the direct tunnelling barrier and the Schottky barrier height are determined. Lines are the corresponding fittings. The fitting parameters are shown in Supplementary Table 1.

The thermal behaviour of LRS and HRS are investigated by checking the evolution of *I*−*V* curves at different temperatures between 295 K and 440 K as shown in Supplementary Fig. 9. The parabolic LRS *I*−*V* curves show weak temperature dependence, a feature of direct tunnelling. The fitting as shown in the inset according to Equation 1 in the maintext gives a barrier height of ~ 0.41 eV for both interfaces at room temperature, which is consistent with that of the device investigated in the main text. Whereas *I*−*V* data of HRS shows an obvious Schottky diode behaviour with the forward current increasing exponentially with the voltage bias. Different with that of

LRS, current of HRS increases with increasing temperature, showing a strong thermal activation behaviour. In order to obtain the Schottky barrier height, we rewrite Equation 2 in the maintext for Schottky emission here,

$$Ln\ \frac{J}{T^2} = Ln\ A^{**} - \frac{\phi_s}{k_B T} + \frac{qV}{nk_B T}, \quad (4)$$

According to this equation, Ln(J / T^2) vs V should show a linear behaviour at different temperatures which was actually observed as shown in Supplementary Fig. 9c. The intercept of these linear fittings gives,

$$F(T) = Ln\ A^{**} - \frac{\phi_s}{k_B T}, \quad (5)$$

By representing $F(T)$ vs. $1/T$ as shown in Supplementary Fig. 9d we could get the Schottky barrier height ϕ_S, which is ~ 0.33 eV. The effective Richardson constant A^{**} is ~ 0.006 A m^{-2} K^{-2}. From which we could get an average tunnelling barrier height ϕ_T of 0.53 eV, which is ~ 0.1 eV larger than the direct tunnelling barrier of 0.41 eV for LRS. The thermionic emission fitting in the reverse direction according to Supplementary Equation 6 gives similar results as that of the forward bias and both of them were summarized in Supplementary Table 1. Generally, the obtained low value of effective Richardson constant, of the order of 0.01−0.1 A m^{-2} K^{-2} with some variations from device-to-device, originates from the direct tunneling of electrons through the SBFO ultrathin layer.

$$Ln\ \frac{J}{T^2} = Ln\ A^{**} - \frac{\phi_s}{k_B T} + \frac{q}{k_B T}\sqrt{\frac{qV}{4\pi d \varepsilon_0 \varepsilon_{op}}}, \quad (6)$$

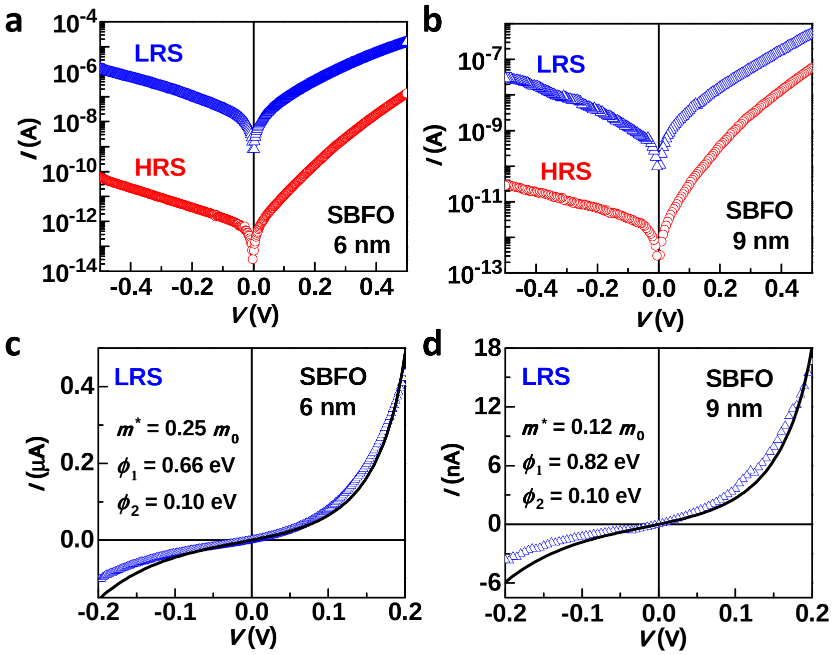

Supplementary Figure 10 | Typical *I-V* characteristics of HRS and LRS for heterostructures with SBFO barrier thickness of 6 nm and 9 nm. (a, b) *I-V* curves; (c, d) Direct tunneling fittings (solid lines) of LRS. The derived fitting parameters such as effective mass and the two interface barriers (listed in the inset) are, however, unreasonable, and the fittings do not match with the experiment data well, suggesting the transport of heterostructures with thicker barrier of 6 nm and 9 nm are no longer dominated by direct tunneling.

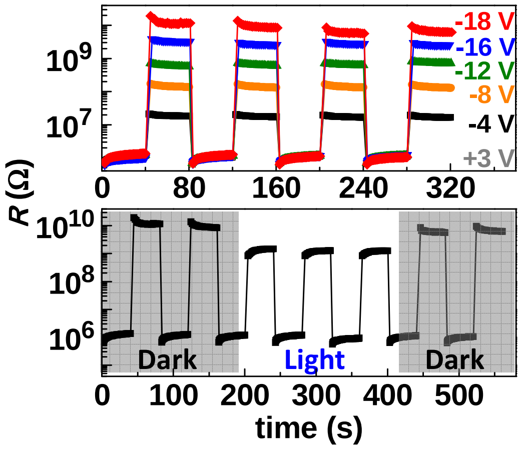

Supplementary Figure 11 | Switching cycling test of NSTO (0.7 wt. %)/SBFO (3 nm)/Pt FTJs. Top, under external voltage pulses of different magnitude in the dark. Bottom, in the dark and under illumination with fixed voltage pulses of −18 V and +3 V.

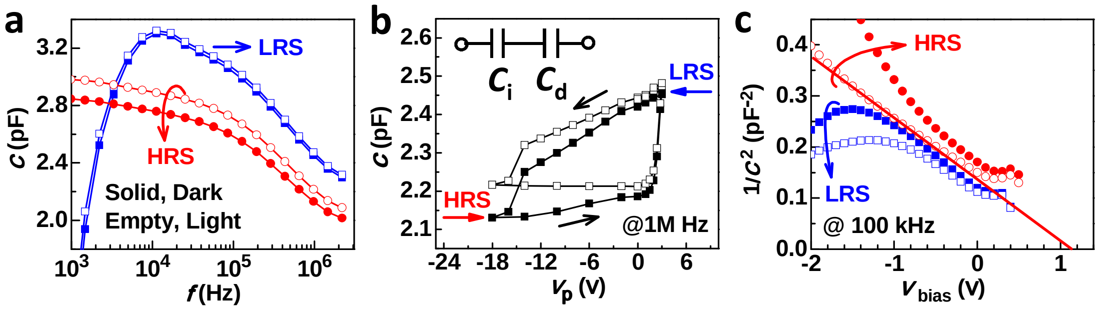

Supplementary Figure 12 | Capacitance controlled by voltage and UV light for NSTO (0.7 wt. %)/SBFO (3 nm)/Pt FTJs. (a) Frequency dependent capacitance measured for typical LRS and HRS after setting by voltage pulse of +3 V and −18 V with pulse width of 1 s. (b) Capacitance at 1 MHz after different voltage pulses. The depletion layer thickness can be estimated from the series capacitance model as shown in the inset. (c) $1/C^2$ vs V for LRS and HRS, respectively. Solid data are obtained in the dark and empty data are obtained under UV light illumination. An alternating voltage signal with amplitude of 0.2 V was used during the measurement.

Quantitative determination of the depletion width of NSTO could be obtained from the capacitance measurement. Total capacitance of the present heterostructures is composed of the depletion capacitance (C_d) in series with the tunnel capacitance (C_i), i.e., $1/C = 1/C_i + 1/C_d$. The formation of the depletion layer in HRS on one hand reduces the total capacitance and on the other hand makes it sensitive to UV light illumination. Both of them are indeed observed as shown in Supplementary Fig. 12a. In the high-frequency regime (> 100 kHz), the total capacitance decreases with increasing frequency, which happens when the electrons or space charges can't keep up with the small signal variation.[3] The abnormal low capacitance at low frequency for LRS is probably due to the high leakage. Accordingly, we focused our capacitance measurements on the high-frequency regime between 10 kHz to 2 MHz. As expected, the capacitance of HRS is smaller than that of LRS. Compared with LRS, light illumination induces an obvious enhancement of capacitance in HRS. This could be understood as light-excited electrons will diffuse toward the NSTO interface and reduce the depletion width. Supplementary Fig. 12b shows the capacitance measured

at 1 MHz as a function of the voltage pulse. The $C-V_p$ loops share the same feature as that of the $R-V_p$ memory loops because both are closely related with the voltage-controlled formation of the depletion layer at the NSTO/BFO interface. Supplementary Fig. 12c shows the $1/C^2-V$ curves of HRS and LRS in the dark and under light illumination. The built-in voltage V_{bi} and the space charge density N_d in the depletion region of NSTO for HRS could be estimated from the following equation,[9]

$$1/C^2 = (2n^2 / q\varepsilon_0\varepsilon_s N_d)(V_{bi} - V/n) \quad (7)$$

where n is the ideality factor (~ 2 for HRS obtained from $I-V$ curves in Fig. 4b). This lead to V_{bi} ~ 0.56 eV for HRS. N_d extracted from the fitting is ~ 1.5 x10^{20} cm^{-3} for HRS, which is consistent with that of 0.7 wt. % Nb: STO (~ 10^{20} cm^{-3}).

The depletion width of NSTO at HRS can be estimated by $qN_dW_d = \frac{2d\varepsilon_s}{2d\varepsilon_s + W_d\varepsilon_F}P$,[10] if we assume that the polarization is fully compensated by the space charge N_d. Using the above acquired N_d of 10^{20} cm^{-3}, ε_s of 200 for NSTO, ε_F of 45 for SBFO (from the bulk measurement) and a reasonable P value of 15 μC cm^{-2} for 3 nm SBFO film in the $[001]_{pc}$ direction,[11] W_d is estimated to be 7.3 nm, which is close to that estimated from the $I-V$ data (8.5 nm).

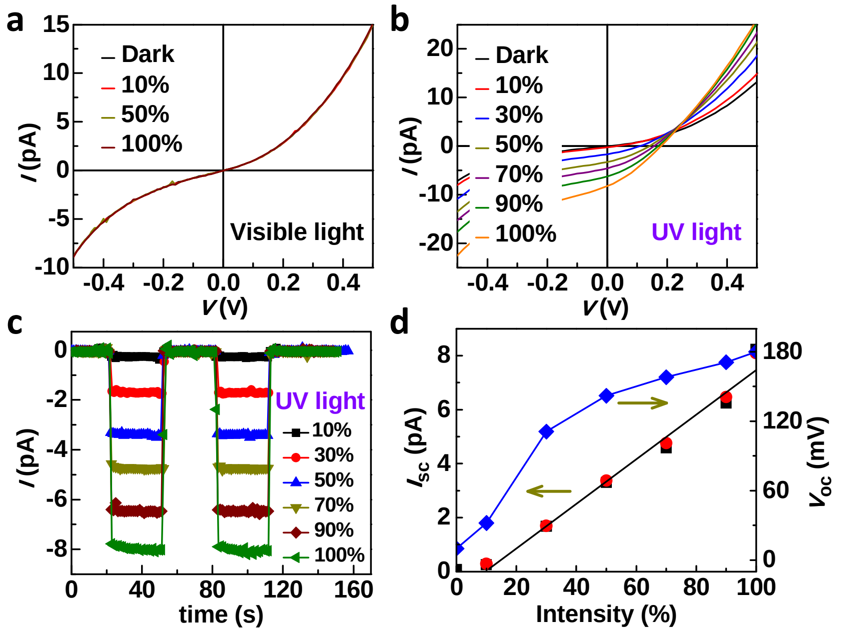

Supplementary Figure 13 | Photovoltaic effect for NSTO (0.7 wt. %)/SBFO (3 nm)/Pt FTJs. (a) Under visible light illumination (Max-302, 385−740 nm, maximum intensity = 1.5 W cm^{-2}) and (b) (c) (d) under UV light illumination (Max-303 250−385 nm, maximum intensity = 60 mW cm^{-2}). (a) (b) *I*−*V* curves. (c) Light intensity dependent photocurrent *vs.* time in the dark and under illumination. (d) Light intensity dependent I_{sc} and V_{oc} obtained from (b) and (c). The band gap of SBFO is ~ 2.7 eV (~ 460 nm), which is within visible light range. The photovoltaic (PV) effect has been previously observed in $BiFeO_3$ single crystals and thick films,[12,13] however we do not observe the PV effect when exposing the devices under visible light (385−740 nm) with intensity of up to 1.5 W cm^{-2} (Supplementary Fig. 13a). Whereas considerable PV effect is observed when the UV light (250−385 nm) with intensity of 6−60 mW cm^{-2} was applied (Supplementary Figs. 13b and 13c). The bandgap of Nb-doped $SrTiO_3$ is ~ 3.2 eV and the corresponding band-to-band excitation wavelength is 388 nm, which is just on the edge of this UV light range. We thus conclude that the active layer in the present devices is mainly the NSTO substrate. As shown in Supplementary Figs. 13 b-d, both I_{sc} and V_{oc} increase with increasing light intensity. This is expected as a stronger light intensity can generate more electron-hole pairs which are separated by the internal electric field. No saturation of I_{sc} and V_{oc} are observed up to 60 mW cm^{-2}, which should be due to the relative low

transmittance of the top electrode Pt 15nm/ITO 350 nm (less than 10% in the UV region, not shown).

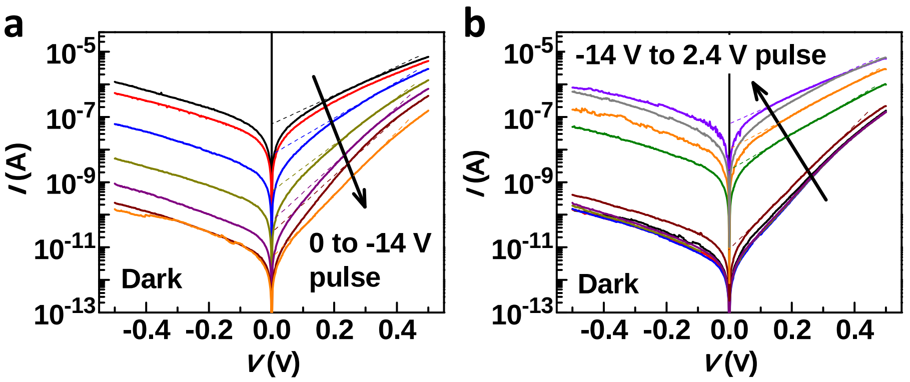

Supplementary Figure 14 | Resistance-dependent *I-V* curves in the dark for NSTO (0.7 wt. %)/SBFO (3 nm)/Pt FTJs. (a) after the successive voltage pulses of 0, −2, −4, −6, −8, −10, and −14 V; (b) after the successive voltage pulses of −14, −10, −6, −2, 0, 1, 1.5, 2, 2.2, and 2.4 V. The device was first set to LRS by a +2.4 V pulse before the measurement. The dotted lines are the Schottky thermionic fittings.

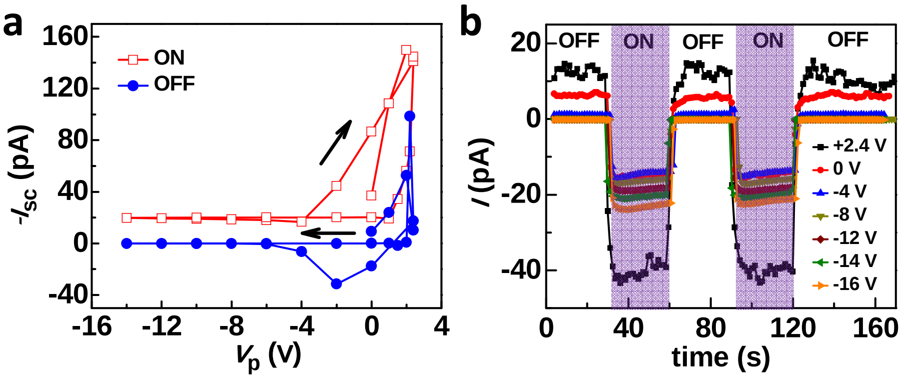

Supplementary Figure 15 | The short circuit current I_{sc} at different resistance states. (a) I_{sc} data obtained from *I-V* curves in Fig. 4, and (b) I_{sc} vs. time with UV light being turned off and on for NSTO (0.7 wt. %)/SBFO (3 nm)/Pt FTJ. As can be seen, I_{sc} vs. V_p also shows a hysteresis behaviour. This however is suspicious as considerable current response at zero voltage bias also appears in the dark. I_{sc} as a function of time with UV light being turned on and off (Supplementary Fig. 15b) further illustrates this point. An obvious current response exists even in the dark for

LRS obtained by applying +2.4 V pulse. This is due to the considerable bigger conductance in LRS (~ 4 orders larger than that of HRS). Thereby a small voltage fluctuation could lead to a considerable bigger current fluctuation in LRS than that of HRS. Except this uncertainty for LRS, it is seen that I_{sc} is around 20 pA for different resistive states, and it has a gradual increase (from 15 pA to 23 pA) with increasing the resistive values by applying voltage pulses from −4 V to −16 V. This is qualitatively consistent with the picture of the enhanced charge depletion layer formed at the NSTO surface induced by the negative electric field. As has been discussed,[13] an enhancement of depletion layer thickness would lead to an increase of the photocurrent.

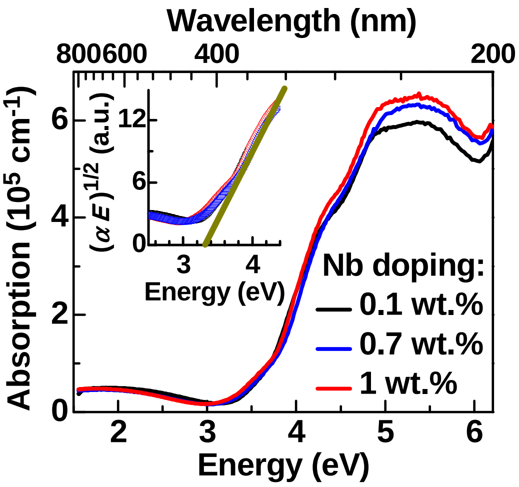

Supplementary Figure 16 | Absorption coefficients of NSTO (100 nm) films with different Nb doping levels (0.1, 0.7, 1 wt.%). Inset, plot of $(\alpha E)^{1/2}$ *vs* E near the absorption edge, from which an indirect band gap of ~ 3.3 eV was derived.

Supplementary Table 1. The fitting parameters derived from the *T*-dependent *I*−*V* data of LRS and HRS.

LRS	m^*=0.9 m_0		ϕ_T = 0.41 eV
HRS, Forward	n = 3-4	ϕ_s= 0.33 eV	A^{**}=0.006 A m^{-2} K^{-2}, & ϕ_T = 0.53 eV
HRS, Reverse	ε_{op} = 7.4<br>ε_{op} = 6 [6]	ϕ_s= 0.46 eV	A^{**}=0.09 A m^{-2} K^{-2}, & ϕ_T = 0.40 eV

Supplementary References

1. Kan, D., Anbusathaiah, V., & Takeuchi, I. Chemical Substitution-induced ferroelectric polarization rotation in $BiFeO_3$. *Adv. Mater.* **23**, 1765–1769 (2011).
2. Khomchenko, V. A. *et al.* Effect of Sm substitution on ferroelectric and magnetic properties of $BiFeO_3$. *Scripta Mater.* **62**, 238–241 (2010).
3. Sze, S. M. & Ng, K. K. *Physics of Semiconductor Devices* 3rd edn (Wiley, 2007).
4. Pintilie, L., Boerasu, I., Gomes, M. J. M., Zhao, T., Ramesh, R. & Alexe, M. Metal-ferroelectric-metal structures with Schottky contacts. II. Analysis of the experimental current-voltage and capacitance-voltage characteristics of Pb(Zr, Ti)O_3 thin films. *J. Appl. Phys.* **98**, 124104 (2005).
5. Pintilie, L., Vrejoiu, I., Hesse, D., LeRhun, G. & Alexe, M. Ferroelectric polarization-leakage current relation in high quality epitaxial Pb(Zr,Ti)O_3 films. *Phys. Rev. B* **75**, 104103 (2007).
6. Wunderlich, W., Ohta, H. & Koumoto, K. Enhanced effective mass in doped $SrTiO_3$ and related perovskites. *Physica B* **404**, 2202–2212 (2009).
7. Reinle-Schmitt, M. L. *et al.* Tunable conductivity threshold at polar oxide interfaces. *Nat. Commun.* **3**, 932 (2012).

8. Van der Berg, R. A., Blom, P. W. M., Cillessen, J. F. M. & Wolf, R. M. Field dependent permittivity in metal-semiconducting $SrTiO_3$ Schottky diodes. *Appl. Phys. Lett.* **66**, 697–699 (1995).
9. Mikheev, E., Hoskins, B. D., Strukov, D. B. & Stemmer, S. Resistive switching and its suppression in Pt/Nb:$SrTiO_3$ junctions. *Nat. Commun.* **5**, 3990 (2014).
10. Wen, Z., Li, C., Wu, D., Li, A. & Ming, N. Ferroelectric-field-effect-enhanced electroresistance in metal/ferroelectric/semiconductor tunnel junctions. *Nat. Mater.* **12**, 617–621 (2013).
11. Maksymovych, P. *et al.* Ultrathin limit and dead-layer effects in local polarization switching of $BiFeO_3$. *Phys. Rev. B* **85**, 014119 (2012).
12. Choi, T., Lee, S., Choi, Y. J., Kiryukhin, V. & Cheong, S. W. Switchable ferroelectric diode and photovoltaic effect in $BiFeO_3$. *Science* **324**, 63–66 (2009).
13. Guo, R. *et al.* Non-volatile memory based on the ferroelectric photovoltaic effect. *Nat. Commun.* **4**, 1990 (2013).
